# Mechanical strength of antibiotic-loaded PMMA spacers in two-stage revision surgery

**DOI:** 10.1186/s12891-022-05895-5

**Published:** 2022-10-29

**Authors:** Andre Lunz, Kevin Knappe, Georg W. Omlor, Mareike Schonhoff, Tobias Renkawitz, Sebastian Jaeger

**Affiliations:** 1 Clinic for Orthopedics, Center for Orthopedics, Trauma Surgery and Spinal Cord Injury, Heidelberg University Hospital, Schlierbacher Landstrasse 200a, 69118 Heidelberg, Germany; 2 Laboratory of Biomechanics and Implant Research, Center for Orthopedics, Trauma Surgery and Spinal Cord Injury, Heidelberg University Hospital, Schlierbacher Landstrasse 200a, 69118 Heidelberg, Germany

**Keywords:** Periprosthetic joint infection, Two-stage revision, PMMA bone cement, dALBC, Spacer

## Abstract

**Background:**

Antibiotic-loaded polymethylmethacrylate (PMMA) bone cement spacers provide high local antibiotic concentrations and patient mobility during the interim period of two-stage revision for periprosthetic joint infection (PJI). This study compares mechanical characteristics of six dual antibiotic-loaded bone cement (dALBC) preparations made from three different PMMA bone cements. The study`s main objective was to determine the effect of time and antibiotic concentration on mechanical strength of dALBCs frequently used for spacer fabrication in the setting of two-stage revision for PJI.

**Methods:**

A total of 84 dual antibiotic-loaded bone cement specimens made of either Copal spacem, Copal G + V or Palacos R + G were fabricated. Each specimen contained 0.5 g of gentamicin and either 2 g (low concentration) or 4 g (high concentration) of vancomycin powder per 40 g bone cement. The bending strength was determined at two different timepoints, 24 h and six weeks after spacer fabrication, using the four-point bending test.

**Results:**

Preparations made from Copal G + V showed the highest bending strength after incubation for 24 h with a mean of 57.6 ± 1.2 MPa (low concentration) and 50.4 ± 4.4 MPa (high concentration). After incubation for six weeks the bending strength had decreased in all six preparations and Palacos R + G showed the highest bending strength in the high concentration group (39.4 ± 1.6 MPa). All low concentration preparations showed superior mechanical strength compared to their high concentration (4 g of vancomycin) counterpart. This difference was statistically significant for Copal spacem and Copal G + V (both *p* < 0.001), but not for Palacos R + G (*p* = 0.09).

**Conclusions:**

This study suggests that mechanical strength of antibiotic-loaded PMMA bone cement critically decreases even over the short time period of six weeks, which is the recommended interim period in the setting of two-stage revision. This potentially results in an increased risk for PMMA spacer fracture at the end of the interim period and especially in patients with prolonged interim periods. Finally, we conclude that intraoperative addition of 4 g of vancomycin powder per 40 g of gentamicin-premixed Palacos R + G (Group D) is mechanically the preparation of choice if a dual antibiotic-loaded bone cement spacer with high antibiotic concentrations and good stability is warranted. In any case the written and signed informed consent including the off-label use of custom-made antibiotic-loaded PMMA bone cement spacers must be obtained before surgery.

## Background

Total hip replacement is considered the operation of the last century in orthopedic surgery and its numbers and numbers of total knee replacements are further rising every year [1, 2]. Based on large register data, periprosthetic joint infection (PJI) is one of the most common causes for revision surgery [3, 4]. Especially a chronic PJI is a very challenging situation for both surgeon and patient, in which a two-stage procedure is still considered the gold standard treatment option [5–7]. During first-stage surgery the infected implant is removed, a thorough debridement conducted, and a spacer can be inserted. After an interim period, second-stage surgery is performed with removal of the spacer and implantation of a new endoprosthesis. Current guidelines recommend a standard interim period of 6 weeks for most chronic PJIs but according to numerous recent studies the interim period often lasts around 12 weeks [8–14]. In the setting of two-stage revision a broad variety of different static and articulating polymethylmethacrylate (PMMA) bone cement spacers have been described in the literature [15]. According to numerous studies with different spacer designs their most common mechanical complications are dislocations with a varying rate of 3–19% and spacer fractures with a varying rate of 2–24% [16–21]. To increase mechanical strength and thereby successfully reduce the rate of spacer fractures many institutions use a bone cement-covered metal-endoskeleton for reinforcement [17, 22, 23]. For these and for most PMMA spacers without a reinforcement dual antibiotic-loaded bone cement (dALBC) is used more and more frequently to achieve high local antibiotic concentrations for a wide range of causative agents. But at the same time, the reduced mechanical strength of dALBC can become a source of failure, especially in spacer designs without an additional endoskeleton. Nevertheless, most surgeons allow their patients at least partial weight bearing of the affected joint during the interim period and therefore avoid prolonged patient immobilization [23]. To the best of our knowledge, there is insufficient data to state whether dALBC spacers with or without an endoskeleton reinforcement provide the mechanical durability to allow partial or full weight bearing during the interim period. In addition, most PMMA bone cements are intraoperatively loaded with different amounts of antibiotics to increase antibiotic elution. In these scenarios the surgeon can become responsible for any liability claims, if a written and signed informed consent about an off-label use was not obtained. A more expensive but supposedly legally safer alternative is using a commercially premixed dALBC (e.g., Copal G + V) to avoid manual addition of antibiotics. Since recently another option is available, a bone cement (Copal spacem) specifically developed for spacer fabrication [24]. The powder of Copal spacem includes calcium carbonate particles, which serve as both contrast agent and biodegradable porogen, and no antibiotics are premixed, allowing the orthopedic surgeon to add any pathogen-adjusted antibiotics. A fourth option is provided by commercially available totally prefabricated PMMA spacers. These ready-to-use spacers are less popular as they do not allow to customize local antibiotic therapy, adjust the size, or reinforce the spacers with an endoskeleton. To the best of our knowledge, only little data is available about biomechanics of custom-made dALBCs [25, 26]. Therefore, we compared the mechanical bending strength of six different dALBCs made from three frequently used PMMA bone cements (Copal spacem, Copal G + V, Palacos R + G) and loaded with two different combinations of vancomycin and gentamicin powder as this combination is considered very effective against most PJI-causing pathogens [14].

Our main study objective was to determine the effect of time and antibiotic concentration on mechanical strength of dALBCs frequently used for spacer fabrication in the setting of two-stage revision for PJI. Furthermore, the goals of our study were to identify which dALBC preparations fulfill the minimal mechanical requirements for use in total joint replacement [27, 28] after an incubation for 24 h and six weeks, and whether commercially premixed dALBCs or bone cements specifically developed for spacer fabrication achieve superior mechanical characteristics compared to hand-made dALBC from a standard PMMA bone cement.

## Methods

### Specimen preparation and cementation

A total of six different preparations (Group A-F; Table 1) were made from three different PMMA bone cements. We classified for each bone cement a low (2 g vancomycin) and a high (4 g vancomycin) concentration group according to the total amount of vancomycin powder per 40 g cement powder. A total of 84 specimens were fabricated and tested at two different time points. Copal spacem (Heraeus Medical, Wehrheim, Germany) contains 33.7 g polymethylmethacrylate, 6.0 g calcium carbonate, and 0.3 g dibenzoyl peroxide in a 40 g packaging unit. Palacos R + G (Heraeus Medical, Wehrheim, Germany) contains 0.5 g premixed gentamicin. To produce equal gentamicin loading, 0.5 g of gentamicin sulfate (GENAXXON bioscience, Ulm, Germany) was added to the powder of Copal spacem. Then 2 g or 4 g of vancomycin hydrochloride (Hikma Pharmaceuticals, London, UK) were added to the powder of Copal spacem and Palacos R + G. The third bone cement used was Copal G + V (Heraeus Medical, Wehrheim, Germany). A 40 g packaging contains 0.5 g gentamicin and 2 g vancomycin. We used this bone cement in an unmodified version and in a custom-made preparation with additional 2 g vancomycin hydrochloride (Hikma Pharmaceuticals, London, UK).

**Table 1:** Antibiotic ratio of the six dual antibiotic-loaded bone cement (dALBC) preparations

Group	PMMA bone cement	Premixed gentamicin	Manually added gentamicin	Total amount of gentamicin	Premixed vancomycin	Manually added vancomycin	Total amount of vancomycin
A	**Copal spacem**	0	0,5 g	**0,5 g**	0	2 g	**2 g**
*B*	***Copal spacem***	*0*	*0,5 g*	***0,5 g***	*0*	*4 g*	***4 g***
C	**Palacos R + G**	0,5 g	0	**0,5 g**	0	2 g	**2 g**
*D*	***Palacos R* + *G***	*0,5 g*	*0*	***0,5 g***	*0*	*4 g*	***4 g***
E	**Copal G + V**	0,5 g	0	**0,5 g**	2 g	0	**2 g**
*F*	***Copal G* + *V***	*0,5 g*	*0*	***0,5 g***	*2 g*	*2 g*	***4 g***

From each of the three PMMA bone cements two different preparations were tested, a low concentration with 2 g of vancomycin (Group A, C and E;) and a high concentration preparation with 4 g of vancomycin (Group B, D and F; all written in cursive letters)

**Fig. 1:**
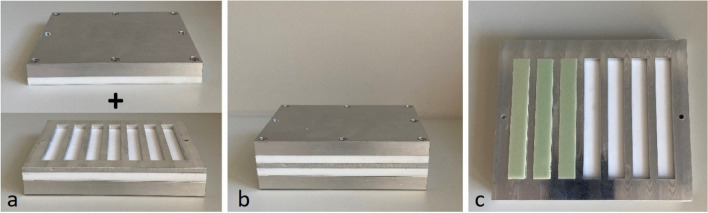
Mold used to form predefined rectangular specimens. **a** and **b** The mold consists of a bottom plate, a PTFE (polytetrafluoroethylene) plate, a polyester film, and a molds plate. It is closed by another PTFE plate and a top plate. **c** By applying PMMA bone cement into the molds plate before closing it, predefined rectangular specimens are being formed according to ISO 5833:2002

Manual antibiotic loading was performed following the recommendations by Kuhn et al. [29]. The powder of the added antibiotics was thoroughly ground in a mortar and then successively added to the powder of the bone cement while stirring. Each cementing procedure strictly followed the manufacturer’s instructions. All cement-mixing procedures were performed without vacuum at a room temperature of 23 ± 1 °C and humidity of at least 40%. Exactly 60 s after bone cement polymerization was started, the bone cement was applied into the mold using a cement gun. The mold was constructed to form 7 predefined rectangular blocks with the size of 3.3 × 10 × 75 mm for each spacer group (ISO 5833:2002) [27]. To smoothen the surfaces, the bottom and the top plate of the mold were covered with a heat-stable polyester film (Tartan transparency film 901, 3 M, Saint Paul, MN, USA) in combination with a PTFE (polytetrafluoroethylene) plate (Fig. 1). Then, the molds were clamped for an hour to achieve complete curing of the bone cement. [30].

### Geometry and radiopacity

**Fig. 2:**
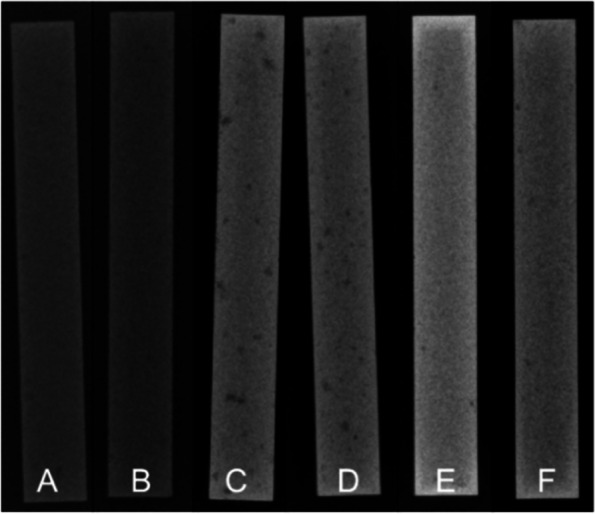
Exemplary radiographs of a specimen from each of the six groups

After polymerization for one hour inside the clamped mold, all specimens were carefully removed and examined radiologically for irregularities like air inclusions (Fig. 2). Then, according to ISO 5833:2002, the surface of all specimens was carefully smoothened and measured to fulfill the geometry requirements of a rectangular block with a length of 75 ± 0.2 mm, a width of 10 ± 0.2 mm, and a total thickness of 3.3 ± 0.2 mm [27]. Each measurement was performed three times with an accuracy of ± 0.1 mm. An average value was calculated for the respective sample geometry.

### Bending strength testing

**Fig. 3:**
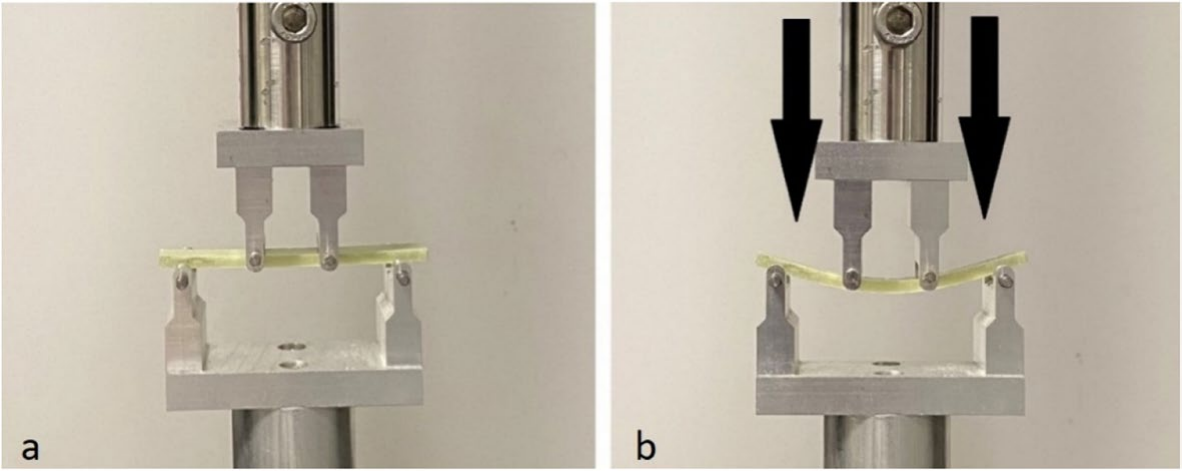
Four-point bending test as described in ISO 5833:2002 and in ISO 16402:2008. Material testing machine (Zwick/Roell Z005, Ulm, Germany) with inserted specimen **a** at the beginning without any force loading and **b** with increasing force loading onto the tested specimen

Each dALBC specimen was stored separately in 40 ml phosphate-buffered saline (PBS) with pH 7.4 at 37 °C. We performed the bending strength testing at two timepoints. According to ISO 5833:2002 and ISO 16402:2008, the first point in time was 24 ± 2 h (24-h) after specimen preparation. The second point in time was after incubation for six weeks (6-w) in PBS, which is equivalent to six weeks in vivo. A series of seven specimens was tested at each timepoint for each group. According to ISO 5833:2002, the specimen`s size, defined as a rectangular block with a length of 75 ± 0.2 mm, a width of 10 ± 0.2 mm, and a total thickness of 3.3 ± 0.2 mm, and the testing conditions, with room temperature of 23 ± 1 ◦C and dry specimens, were satisfied. The bending strength was determined using a four-point bending test as described in ISO 5833:2002 and in ISO 16402:2008 [27, 28]. The four-point bending test was carried out with a material testing machine (Zwick/Roell Z005, Ulm, Germany) and a crosshead speed of 5 mm/min (Fig. 3). The force on the central loading points was increased, starting from zero until the specimen broke. The deflection of the specimen was recorded as a function of the applied force. For each tested specimen, the bending strength was calculated using the following equation: B = 3Fa / bh^2^, with F (in N) as the force at break, b (in mm) as the average measured width of the specimen, h (in mm) as the average thickness of specimen, and a (in mm) as the distance between the inner and outer loading points. According to the ISO 5833:2002, the distance between the outer and inner loading points was set to 20 mm.

### Statistical analysis

All descriptive data is presented as the arithmetic mean, standard deviation and minimum and maximum. The Shapiro–Wilk test was performed to confirm normal distribution of the data. Next the Levene's test confirmed equality of variances. Therefore, the one-way ANOVA with post hoc analysis for independent variables was applied. To compare the groups, an ANOVA with a Bonferroni correction was used. The level of significance was set at *P* < 0.05 for all statistical tests. The statistical analyses were performed using the software “SPSS” (version 27.0; IBM Inc., Armonk, NY, USA).

## Results

**Fig. 4:**
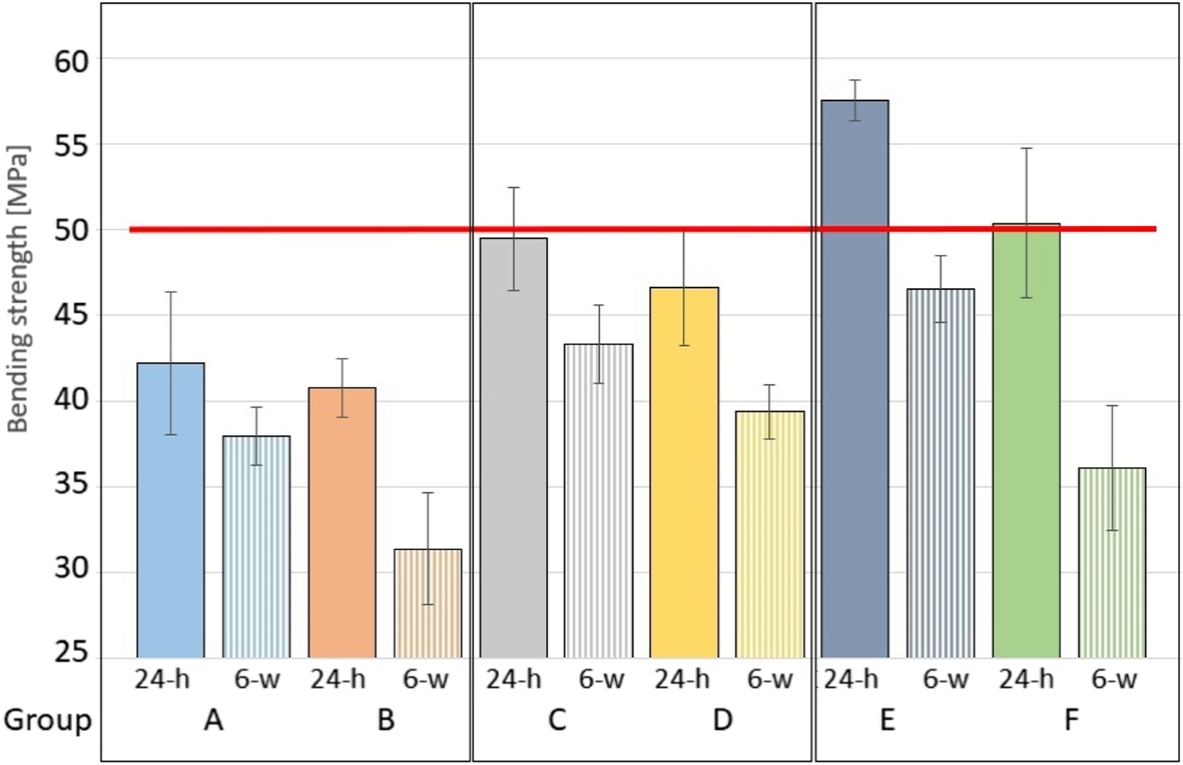
Mean bending strength [MPa] of the six groups (**A-F**) after incubation for 24 h (24-h) and six weeks (6-w). The minimal requirement of 50 MPa according to ISO 5833:2002 is marked with a red horizontal line [27]. Preparations from Group E and F have surpassed this threshold after incubation for 24 h with a mean bending strength of 57.6 ± 1.2 MPa and 50.4 ± 4.4 MPA, respectively

Antibiotics were manually administered to the three different bone cements to create a low (2 g vancomycin + 0.5 g gentamicin) and a high (4 g vancomycin + 0.5 g gentamicin) antibiotic concentration group for each of the three bone cements. In total six different dual antibiotic loaded bone cement (dALBC) groups were created with each group consisting of 14 identical predefined rectangular specimens. All 84 specimens fulfilled the requirements of ISO 5833:2002 [27] and the four-point bending test was performed after incubation of the specimens for 24 h and six weeks (Fig. 4).

First, we tested seven specimens from each of the six groups after incubation in PBS for 24-h. The preparations made from Copal spacem (Group A and B) showed the lowest bending strength with a mean of 42.2 ± 4.2 MPa and 40.8 ± 1.7 MPa, respectively, while preparations made from Copal G + V (Group E and F) achieved the highest results with a mean of 57.6 ± 1.2 MPa and 50.4 ± 4.4 MPa, respectively. Spacers made from Palacos R + G (Groups C and D) showed a bending strength of 49.5 ± 3 MPa and 46.6 ± 3.4 MPa, respectively.

After incubation for 6 weeks in PBS the four-point bending test was repeated in the same way. Spacers made from Copal G + V (Group E and F) showed the largest decrease in bending strength with a mean bending strength of 46.5 ± 2 MPa (mean difference of 11.1 MPa) and 36.1 ± 3.7 MPa (mean difference of 14.3 MPa), respectively. Preparations made from Copal spacem (Group A and B) showed a mean bending strength of 38 ± 1.7 MPa (mean difference of 4.2 MPa) and 31.4 ± 3.3 MPa (mean difference of 9.4 MPa), respectively, while preparations with Palacos R + G (Groups C and D) showed a mean of 43.4 ± 2.3 MPa (mean difference of 6.1 MPa) and 39.4 ± 1.6 MPa (mean difference of 7.2 MPa), respectively.

Two preparations (Group E and F) surpassed the minimum requirement of 50 MPa according to ISO 5833:2002 and ISO 16402:2008 after incubation for 24 h. None of the tested preparations passed the minimum requirement after incubation for six weeks.

**Fig. 5:**
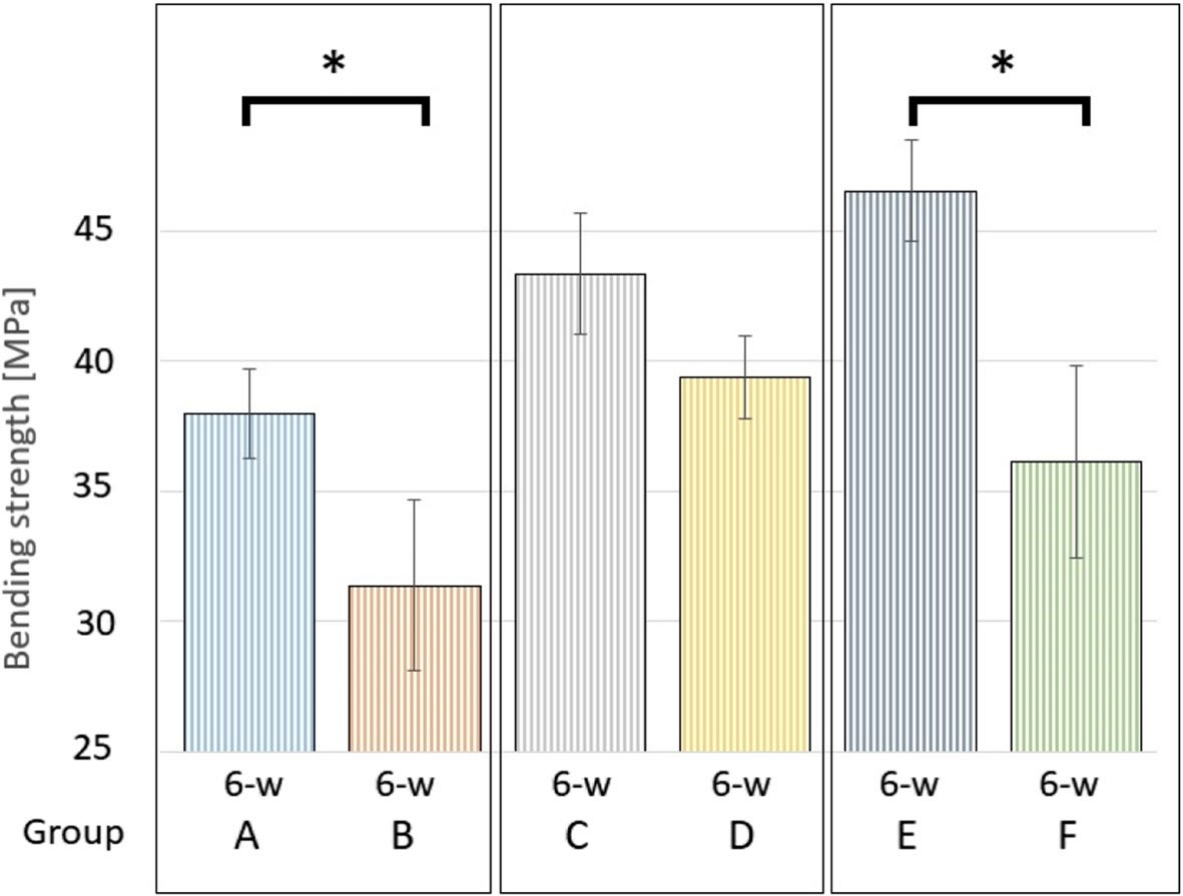
Mean bending strength [MPa] after incubation for six weeks (6-w). Statistically significant differences between specimens fabricated from the same PMMA bone cement (low concentration vs. high concentration group) are marked with an asterisk (*)

**Fig. 6:**
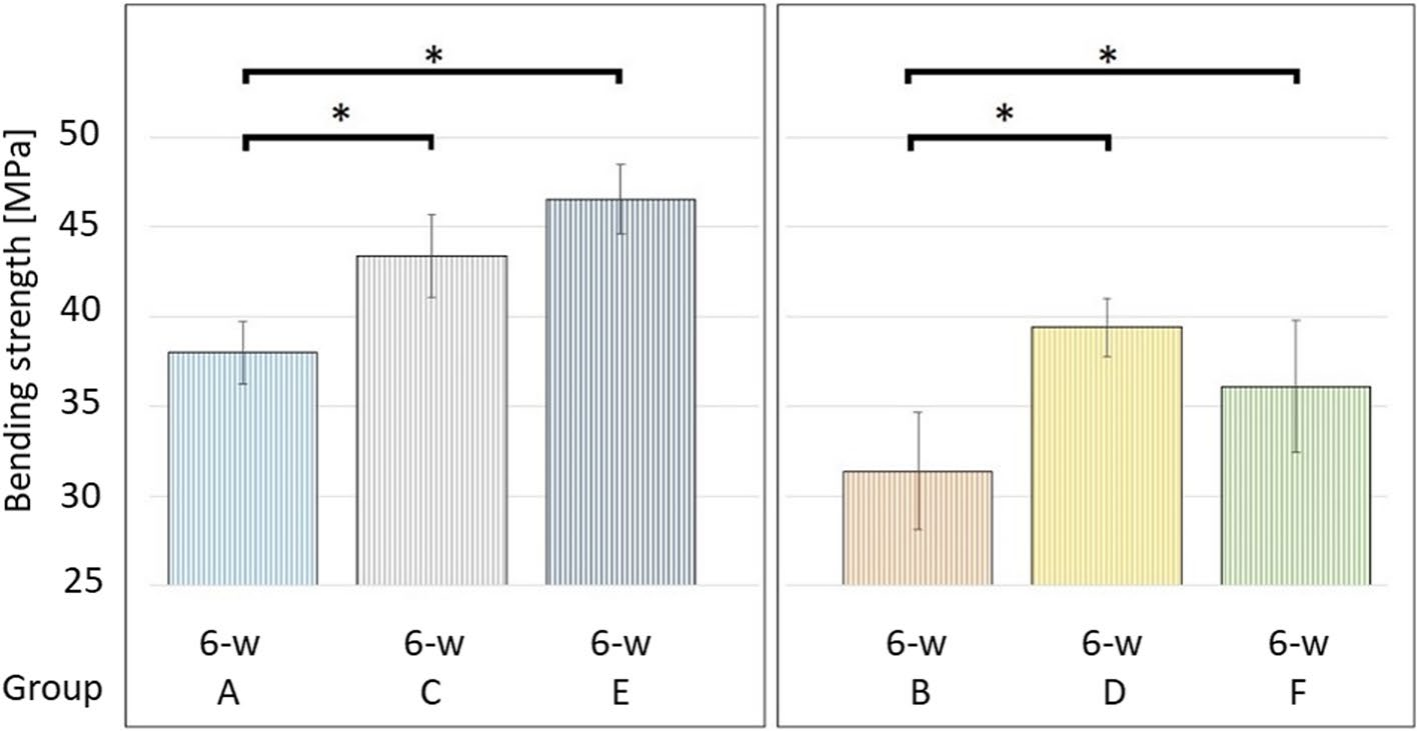
Mean bending strength [MPa] after incubation for six weeks (6-w). “Low” concentration preparations with 2 g of vancomycin (Group **A**, **C** and **E**) are shown on the left and “high” concentration preparations with 4 g of vancomycin (Group **B**, **D** and **F**) on the right. Statistically significant differences are marked with an asterisk (*)

A one-way ANOVA was performed to compare the bending strength between all groups after incubation for six weeks and a statistically significant difference was found (F (5, 36) = 30.96, *p* < 0.001). Therefore, a post hoc analysis was performed for further analysis. We compared the bending strength between the low and high concentration preparations of the same bone cements and found a statistically significant difference between Group A and B (Copal spacem, *p* < 0.001) and Group E and F (Copal G + V; *p* < 0.001) but no significant difference between Group C and D (Palacos R + G; *p* = 0.09), as illustrated in Fig. 5. Then, we compared all preparations with 2 g of vancomycin (low concentration group) and a statistically significant difference was found between Group A and C (*p* = 0.005) and Group A and E (*p* < 0.001), but no significant difference was found between Group C and E (*p* = 0.377). Finally, we compared all preparations with 4 g of vancomycin (high concentration group) and a statistically significant difference was found between Group B and D (*p* < 0.001) and Group B and F (*p* = 0.02), but no significant difference was found between Group D and F (*p* = 0.328), as shown in Fig. 6.

## Discussion

Two stage revision surgery remains the most effective treatment option for most chronic PJIs. After removal of the infected implant most surgeons use either an antibiotic loaded PMMA bone cement spacer with or without an endoskeleton reinforcement for the interim period [8, 31]. These spacers provide high local antibiotic concentrations and mechanical stability of the affected joint [32]. Especially articulating spacers can even provide a good range of motion and therefore maintain patient’s mobility [9, 23, 33, 34]. To avoid spacer-related complications like dislocation, peri-spacer-prosthetic fracture, and fracture of the spacer many departments recommend their patients to only perform partial-weight bearing during the spacer-period. But not all patients are able to follow this instruction, as especially multimorbid and old patients cannot walk on crutches to relieve their operated joint. This results in either patient immobilization or full weight bearing with the spacer. While the risk for dislocation is significantly influenced by spacer design, insufficient mechanical strength of a PMMA spacer can lead to a spacer fracture. Both are severe complications usually leading to unplanned revision surgery with exchange of the failed spacer. Especially PMMA hip spacers resulted in high spacer fracture rates of 13–24% [19, 20]. To reduce the risk of spacer fractures many orthopedic surgeons successfully use a metal endoskeleton for reinforcement of the PMMA hip spacers [22, 23, 35]. Disadvantages, however, are higher expenses and potentially a higher risk for microbial contamination of the metal surfaces as they often cannot be completely covered with antibiotic-loaded bone cement. For all other types of spacers, like conventional articulating knee spacers which do not have a metal endoskeleton, the mechanical Achilles’ tendon remains the reduced strength and durability of the dALBC used for their construction [36–38]. Manual intraoperative addition of antibiotics to any PMMA bone cement changes the cement`s and thus spacer’s biomechanical characteristics potentially resulting in a higher risk for mechanical failure due to cement and thus spacer fracture. But manual intraoperative addition of antibiotics still is and has been standard practice in revision surgery for many years. The goal is to increase local antibiotic concentrations considerably above the minimum inhibitory concentration (MIC). The combination of gentamicin and vancomycin is considered very effective against most PJI-causing pathogens and in-vitro testing has shown that addition of these two antibiotics induces synergistic effects [14]. In this study we have fabricated six dual antibiotic loaded bone cement (dALBC) groups from three different bone cements (Copal spacem, Copal G + V, Palacos R + G) with two different antibiotic ratios (low concentration group: 0.5 g gentamicin and 2 g vancomycin; high concentration group: 0.5 g gentamicin and 4 g vancomycin) and compared their mechanical strength at two different points in time. It is important to note that all cement-mixing procedures were performed without vacuum as it is routinely done in the operating theater to increase porosity and therefore antibiotic elution from spacers. Also, the incubation process was performed in a PBS solution to better mimic intraarticular conditions. These alterations have influence on the polymerization process of the bone cement and its porosity leading altogether to a decreased bending strength if compared to vacuum cement-mixing and incubation on air as described in ISO 5833 and the pass mark of 50 MPa [27].

We were able to show that bending strength decreased in all specimens with a longer incubation time in an in-vitro setting. After incubation for six weeks, which is the recommended time between stages for most chronic PJIs [14, 39], none of the tested preparations surpassed the threshold value of 50 MPa. The fact that most original and review studies indicate a mean interim period of around 12 weeks between stages is even more worrisome [10–12]. This prolonged interim period most probably results in a further substantial decrease in mechanical strength of the dALBC resulting in an increased risk for cement fracture. This seems especially relevant for multimorbid patients where second-stage surgery with re-implantation of a new endoprosthesis is often significantly delayed or sometimes even temporary cancelled resulting in interim periods of 4–6 months or more. Therefore, our results suggest that the mechanical strength of the used dALBC for spacer fabrication is insufficient (according to DIN ISO 5833:2002 and ISO 16402:2008 [27, 28]) in most patients undergoing two-stage revision. We further conclude that the risk of cement fractures and thus spacer fractures increases towards the end of the interim period as the mechanical strength of dALBCs decreases. Orthopedic surgeons should therefore aim to keep the interim period as short as necessary if dALBC spacers were used.

Furthermore, our results confirm that intraoperative manual addition of antibiotic powder to any PMMA bone cement decreases its mechanical strength as all preparations from the low concentration group (2 g of vancomycin) showed superior mechanical strength compared to their counterpart (high concentration groups with 4 g of vancomycin). The most likely explanation is that the bone cement`s porosity is increased by adding antibiotic powder to the bone cement. The difference was statistically significant between both preparations made from Copal spacem (Group A and B) and both preparations made from Copal G + V (Group E and F), but not for the two preparations made from Palacos R + G (Group C and D).

To summarize, we have demonstrated that a higher antibiotic concentration can significantly reduce the mechanical strength of dALBCs and potentially results in a higher risk for cement and thus spacer fracture. The orthopedic surgeon must be aware of this conflict between high mechanical stability and high antibiotic concentrations of dALBCs Together, this characteristic and the demonstrated decrease of mechanical strength over time, can help the clinician to make an individual and evidence-based decision for a certain spacer design and dALBC preparation depending on the circumstances of the given case. To reduce the risk of spacer fractures, orthopedic surgeons often have the option to reinforce a PMMA spacer with an endoskeleton and use the most appropriate combination of bone cement and antibiotics. Furthermore, clinicians should try to keep the interim period as short as possible (but as long as necessary) as the mechanical strength of dALBCs decreases significantly over time.

The current study has several limitations. First, we examined rectangular PMMA specimens instead of real PMMA spacers. Second, we measured mechanical strength by testing bending strength by performing a unidirectional force onto the cement specimens. Bending strength is of course not the only mechanical characteristic defining mechanical resilience and the four-point bending test does not resemble reality. But it is a standardized and reproducible method to evaluate the mechanical strength of bone cements [27, 28]. Furthermore, our study was limited to three PMMA bone cements and the antibiotics gentamicin and vancomycin with only two different concentrations tested. Other bone cements with different antibiotics should also be investigated but the tested combinations are the most frequently used ones in clinical practice. In this study all cement-mixing procedures were performed without vacuum and all specimens were incubated in PBS solution despite vacuum-mixing and incubation on air according to ISO 5833:2002 and ISO 16402:2008. But we do not see these alterations as real limitations as we have chosen these conditions on purpose to better mimic intraoperative and real-life conditions. Finally, there are many factors, most importantly spacer design and individual patient anatomy contributing to the risk of mechanical spacer complications. Thus, the demonstrated differences in mechanical strength between different antibiotic-loaded bone cements are of unknown clinical relevance even if statistically significant. But we believe that even in the setting of unknown clinical relevance orthopedic surgeons should know the mechanical characteristics of dALBCs and be aware of the impact on mechanical strength by intraoperatively adding antibiotics to any PMMA bone cement to minimize the risk of mechanical complications. Theoretically, financial aspects could be also taken into consideration if different products achieve similar results. Palacos R + G achieved clearly superior results in comparison to Copal spacem and slightly better or at least similar results in comparison to Copal G + V. But Palacos R + G is substantially less pricy than the other two bone cements.

## Conclusions

To summarize, our results confirm that intraoperative addition of antibiotics significantly decreases mechanical strength of PMMA bone cements and therefore most probably that of PMMA spacers. Furthermore, this study demonstrated that mechanical strength of antibiotic-loaded PMMA bone cements critically decreases even over the short time period of six weeks, which is the recommended interim period in the setting of two-stage revision. This leads potentially to an increased risk for cement fracture and thus for PMMA spacer fracture towards the end of the interim period and especially in patients with prolonged interim periods. Finally, we conclude that intraoperative addition of 4 g of vancomycin powder per 40 g of gentamicin-premixed Palacos R + G (Group D) is mechanically the preparation of choice if a dual antibiotic-loaded bone cement (dALBC) spacer with high antibiotic concentrations and good stability is warranted. In any case the written and signed informed consent including the off-label use of custom-made antibiotic-loaded PMMA bone cement spacers must be obtained before surgery.

## Data Availability

The datasets used and/or analyzed during the current study are available from the corresponding author on reasonable request.

## Abbreviations

24-h: 24-Hours
6-w: 6-Weeks
dALBC: Dual antibiotic-loaded bone cement
MIC: Minimum inhibitory concentration
PBS: Phosphate-buffered saline
PJI: Periprosthetic joint infection
PMMA: Polymethylmethacrylate
PTFE: Polytetrafluoroethylene
